# Hospital and Institutional News

**Published:** 1917-03-10

**Authors:** 


### HOSPITAL AND INSTITUTIONAL NEWS.

### THE ROYAL INFIRMARY, LIVERPOOL.

The report presented at the annual general meeting of the trustees of the Royal Infirmary, Liverpool, discloses what can only be regarded as a most successful year, in spite of war difficulties. The most notable benefaction of the year has been that of Mr. John W. Hughes, an old friend of the infirmary, who gave £4,500 for the special purpose of enabling the committee to buy a piece of land at the corner of Pembroke Place and Ashton Street. The acquisition of this site will make it possible to use to the best advantage the land already belonging to the infirmary, when circumstances permit of the erection of the new nurses' home, which is so urgently needed. As a nucleus of a fund for this building the honorary treasurer has given £500, and the committee express the hope that other friends may follow this fine example. During the year the Rector of Liverpool held a special service in the Parish Church in aid of the infirmary, when the offertory totalled £266; it is hoped that this will become an annual institution. Sir W. P. Hartley, another old friend of the infirmary, gave £1,000 of War Loan to commemorate his seventieth birthday; and Mr. Bickerton, who retires this year from the post of ophthalmic surgeon after thirty years' tenure of it, has presented £500 of War Savings Certificates, the proceeds of which are to be spent in a special manner indicated by him. Mr. Bickerton entered the infirmary as a student forty-three years ago, and was unanimously elected consulting ophthalmic surgeon. Mr. Arthur S. Mather was elected president for the ensuing year in succession to Sir Aubrey Brocklebank. Captain Holford was re-elected honorary treasurer.

### A SURGEON OF THE "DREADNOUGHT."

One of the last surviving members of the hospital ship *Dreadnought* died on February 22 in the person of Mr. Robert Percy Middlemist, who was acting surgeon on the ship in 1865. He was probably a colleague of Mr. Johnson Smith, so long the principal medical officer of the hospital on water and land, who died a few years ago. Mr. Middlemist, after leaving the *Dreadnought*, acted during the cholera epidemic of 1866 as assistant medical officer of health for the St. Giles' District, and at the time of his death had been for some time honorary surgeon to the Royal Masonic Benevolent Institution, the Royal General Theatrical Fund, and the Actors' Benevolent Fund. He qualified in 1860, after carrying out his medical training at King's College Hospital.

### THE GROWTH OF THE PAY-WARD MOVEMENT.

In an interesting speech at the annual court of the Royal Sussex County Hospital, Brighton, Mr. R. Ball Dodson, chairman, said that many suggestions had been made to him for the establishment of pay-wards "as a means of easing the burden of maintenance." He then pointed out that nowadays it was not so much the poverty of the patient as the seriousness of his complaint which called for admission to hospitals. He hoped that the legal difficulties of admitting paying patients to the present building might be overcome if some wards had to be closed for financial reasons, since he was informed that they could then be reopened as pay-wards. Mr. Ball Dodson probably realises better than those who make proposals to him that a pay-ward may be self-supporting, but that it fails of its object if it becomes (as some vainly hope) a source of income, which "to ease the burden of maintenance" apparently implies. Two guineas a week is the normal figure, and pay-wards must be few.

### VOLUNTARY RATIONING OF THE HOSPITAL STAFF.

It is pointed out in a leading article in *Guy's Hospital Gazette* that a calculation contained in a report by a committee of the Royal Society on the food supply of the United Kingdom, taking into consideration the relative number of men, women, and children, shows that 100 men, women, and children are equal to 77 adult units, or "men." This being so, Lord Devonport's figures as applied to a community of adults should be increased to an allowance per head of 4 lb. of flour, $3\frac{1}{5}$ lb. of meat, and 1 lb. of sugar per week, if it is to obtain its fair share of the total food available for the nation. But as it is admitted that the allowance needed by a woman is less than that required by a man, the *Gazette* suggests that four-fifths of the weights quoted would be the fair quantity for a woman. This in a household composed entirely of women would work out as follows: flour $3\frac{1}{5}$ lb., meat $2\frac{1}{2}$ lb., and sugar $12\frac{4}{5}$ oz. As the greater part of the staff of a hospital is composed of women, the calculation on the lines suggested does not provide a much larger allowance than that suggested by Lord Devonport. Whether hospital employees, in view of the nature of their work, are entitled to greater generosity than other folk is another matter.

### TRIBUNALS AND HOSPITAL EMPLOYEES.

Hospital administrators are admittedly put to great straits and many makeshifts owing to the conscription of their able-bodied male employees for the Army; but their difficulties are no greater than those of many other corporations and of individual employers. It is bad policy to take cases which are intrinsically weak officially before the Tribunals; and, what is more, it is unpatriotic. One London hospital, not of large size or of exceptional repute, has just sustained a rebuff from the Law Society Appeal Tribunal by asking for the exemption of an attendant in the bacteriological laboratory, aged thirty-one. The appeal was supported by a solicitor and by a vice-president of the hospital in question; but it failed, as it deserved to. The solicitor was allowed to make several astounding statements, which were pounced upon by members of the Tribunal; we hope and trust that he was misinformed as to the facts. He is reported to have said, for instance, that the man on whose behalf he appeared is "head of the laboratory and responsible for the pathological examinations and the preparation of serums." If this is true, the authorities of the London Homœopathic Hospital deserve the severest censure; we can hardly believe that it is even an approximation to the truth, however. It was also stated, by the vice-president, who associated himself with the appeal, that the employee in question "analyses the blood of a patient . . . and will say whether the evidence is positive or negative." This also, we feel confident, must be inexact; but hospitals which send representatives to these Tribunals have only themselves to blame if they are not represented properly. What weighed with the Tribunal was obviously the evidence that the responsible functions thus described are carried out by a man who is paid fifty shillings a week!

### TUBERCULOUS GERMANS INTERNED IN SWITZERLAND.

In a tuberculosis dispensary journal which seems to have been rather recently begun (*Tuberkulose-Fürsorge Blätter*, November 7, 1916), the well-known specialist, Turban, describes the arrangements made for German tuberculous soldiers interned at Davos. They are stated to number 11 officers and 554 non-commissioned officers, men, and civilian internists; and to have been selected from prisoner camps in France by ten committees of investigation, controlled by a Commission sitting in Lyons. The invalids are accommodated in houses taking no other patients, and allowed daily rations on a scale rather more liberal, especially respecting meat, than that prevalent in Switzerland generally. Their life is regulated by a routine directed by sanitary officers. The medical men entrusted with their treatment are of Swiss nationality, and possess military authority. The German Government pays 8 francs a day for each officer, and 5 francs a day for each man. Some help is given, too, by charity, and the local German Club contributes. The therapeutic results are stated to be good, and the ameliorated or cured cases are drafted off elsewhere to make room for others.

### A WESTERN SEAMEN'S HOSPITAL.

The amount of the work done by the Royal Hamadryad Seamen's Hospital, Cardiff, in relieving the sufferings of distressed seamen during the past year has been greater than ever before, and had it not been for the extra beds supplied by the President, Sir W. J. Tatem, the medical superintendent says the institution could not have accomplished what it has. The in-patients numbered 1,116—or more than double the total treated ten years ago. The accounts show a deficiency of £1,309, and to meet this a special fund is being collected, with satisfactory results. The hospital invested £5,000 in the last War Loan.

### THE PLIGHT OF AN OLD DISPENSARY.

That the number of patients at the Exeter Dispensary continues to remain large while the income received grows smaller, is a curious comment on the public of Exeter, on the effect of the Insurance Act. If that Act were not to make dispensaries more or less superfluous, what cases are sufficiently trivial for the Act to take under its wing? The answer is that the Act, which provided no institutional treatment, also made virtually no provision for married women and children, and from these the patients of dispensaries are mainly drawn. But the Act has hit the dispensaries, because people imagine that they must now be superfluous, and such a fall in the number of patients as has been noticed is attributed to the fewer available "recommends" which accompany a smaller list of subscribers. On March 16 the dispensary celebrates its centenary. Is no one in Exeter intelligent enough to come to the rescue by supporting a campaign for annual subscriptions?

### THE RELATIONS OF HEALTH VISITORS AND MEDICAL MEN.

In one or two instances in Wales, objection has been raised to the visits paid by health visitors under the local authority, and one medical man, in reply to a letter from the Council, has put on record his protest. At the last meeting of the Mumbles District Council, a communication was read from the practitioner in question, Dr. Coverley Veale, stating that he "did object to the infant welfare visitor visiting his patients when he was in attendance, as it might lead to friction. Interference would be bound to arise." Notwithstanding this protest, the Council decided to issue instructions to the nurse to visit all cases where her services are required. With tact and good will on both sides, friction is not likely to arise, and the kind of doctor who suggests that it must is just the sort of man who possesses but little of either quality.

### A CURIOUS ANNOUNCEMENT.

The provision of expert medical attendance, and more especially of high-class hospital accommodation, for persons of moderate means who cannot afford the cost of specialists' fees and nursing-home charges and yet are not so impoverished as to be qualified for charitable treatment in voluntary hospitals, is one of the reforms in our social arrangements which is long overdue and has been many times supported in these columns. On general principles, therefore, any well-conceived project which aims at supplying such a definite want is certain in advance of sympathy and support in The Hospital. A proposal has lately been set on foot to establish a clinic for the deaf in London, at fees within the means of the middle classes; but the manner and method in which this scheme has been announced to the world at large is certainly unfortunate, to say the least of it. A lay newspaper (the *Morning Post*) has been chosen as the vehicle of the announcement in question, which states that the clinic will be "under the control of aural surgeons" who are not named. It may seem a small matter to the public that the ordinary professional journals have not been chosen as the medium for ventilating this suggested clinic; but unless the medical profession has before it full particulars of the organisation of such a clinic, and the names of all those to be associated with it, it is clearly impossible for patients to be recommended to it with the very slightest confidence. The clinic may be perfectly honest, genuine, and well conducted; but only full publicity through normal channels can afford medical men any assurance that this is the case. It is, in these circumstances, all the more deplorable that the paragraph in the *Morning Post* ends with a sentence which is certain to create serious misgiving. "Deaf persons desirous of regaining their hearing are invited to send a postcard to the Organising Secretary" at a certain West-End address. This, of course, is no evidence of bad faith, but merely of unfortunate drafting; but the promoters of the scheme—whoever they may be —can hardly be surprised, if a sentence so reminiscent of the wiles of the unqualified causes the bulk of the profession to view the anonymous "organising secretary" and his propaganda with grave suspicion.

### THE PRUDENTIAL ASSURANCE COMPANY.

Not long ago the public was justifiably impressed by the announcement that the Prudential Assurance Company had taken up in all over twenty-five millions sterling in the new War Loan (including conversions of Exchequer Bonds and the old Loan). Needless to say, no other corporation or individual could come anywhere near this staggering subscription. The annual report of the company, just issued, does not deal with events later than the end of 1916; and therefore the colossal block of War Stock acquired is an additional piece of evidence, were any required, of the mammoth operations and financial stability of the premier British insurance company. The assets of the company, in all its branches, totalled at the end of the year more than ninety-nine millions; probably by this time they reach into nine figures. Nearly seven million pounds' worth of foreign securities were lent to the Treasury during 1916 under the Regulation of Foreign Exchanges schemes. Altogether the report discloses one of the most wonderful years of working that even the Prudential has ever had. It is stated that one-third of our soldiers who have fallen in France have been policyholders in the Prudential, and that about half our Army at the Front is insured in this wonderful company.

### A SUCCESSFUL YEAR AT SWANSEA.

Another £1,000 gift to Swansea Hospital was announced last week, when, in a letter read at the annual meeting, the retiring chairman, Alderman Evan Evans, intimated his intention of giving the sum mentioned for a ward to be named after himself, and also of presenting to the institution a cottage at Langland Bay—one of the South Wales coast beauty spots—for use as a convalescent home for nurses. Swansea Hospital has undoubtedly had a successful year. The income, including special donations, amounted to £22,031. The subscriptions of works and other employees rose from £7,554 to £9,178; and Alexandra Rose Day brought in £1,945; £4,225 has been invested in the War Loan. The debt due to the treasurer has been reduced by nearly £3,000, as compared with last year.

### A DISPUTE ABOUT AN EPIDEMIC AT BOLDON COLLIERY.

Last autumn a considerable outbreak of enteric fever took place at Boldon Colliery, near South Shields. It appears that enteric is endemic at Boldon, possibly through defective housing arrangements which are alleged to exist there. At all events, this epidemic outburst was so considerable that it attracted the attention of the Local Government Board and the Durham County Council, both of which authorities sent an expert representative to investigate. The report based upon this investigation implies a measure of censure on the M.O.H. for the South Shields Rural District, who has presented his employers with a long and detailed reply, which occupies nearly two columns in the local press. Although the M.O.H. is clearly labouring under a sense of injustice, it must be admitted that some of the points which he raises in his defence are very lame, though others seem reasonable enough. The core of the whole matter is probably to be found in his explanation that he is merely a part-time official, paid as such, and cannot afford to devote the whole of his time to public health work. Inasmuch as enteric appears to be endemic in the locality, the solution of the *impasse* would seem to be that the Council should procure themselves a whole-time M.O.H., properly remunerated.

### SUBSTITUTES FOR GLYCERINE.

The shortage of glycerine has led the pharmacist at St. Bartholomew's Hospital to make a series of experiments to find substitutes, which it is hoped will result in a considerable saving in the expenditure on glycerine, whilst at the same time it will assist the Government to preserve for the manufacture of explosives the supply now in hand. Writing to Mr. Thomas Hayes, the clerk to the governors, the pharmacist says: "The Ministry of Munitions having necessarily restricted my supply of glycerine, which is so largely required in the manufacture of explosives, I have had since January 1 to discontinue the manufacture of a popular antiseptic preparation extensively used in this hospital, into the composition of which glycerine largely enters. I have, however, been able to introduce inexpensive substitutes, which I am pleased to find are completely satisfactory. The discontinuance of glycerine in this form alone represents a saving of £200 a year in the casualty and out-patients' departments."

### QUEEN CHARLOTTE'S LYING-IN HOSPITAL

At the recent annual meeting of governors it was reported that there had been a large increase in the number of patients admitted to the wards in 1916—namely, 2,075, as compared with 1,817 in 1915, and 2,058 had been attended at home by the hospital midwives. Of the in-patients 407, and of the out-patients 911, were the wives of sailors and soldiers. In order to cope with the increasing work of the ante-natal department, a new (temporary) out-patient department has been opened adjoining the hospital, and a physician (Miss Frances M. Huxley, M.D.) has been appointed in charge. An infant consultation centre is also being opened, and a physician (Miss Margaret G. Thackrah) has been appointed in charge. The number of emergency cases admitted was greater than ever. Last year there were 183, many of them admitted in a grave condition and suffering from severe complications. The work has been carried on under difficult conditions owing to shortage of nurses. The training-school has, however, well maintained its reputation, and only one candidate out of 113 failed to pass the Central Midwives Board examination. The Ladies' Association continues to give most valuable assistance. In addition to providing all the linen for the hospital to the value of £267, the Association has been able to contribute £500 to the hospital funds. The chairman made a strong appeal for liberal support to enable the committee to pay off the balance (nearly £2,000) of the debt which has accumulated and to meet the cost of maintenance during the current year.

### NIGHT LIGHTS AT SANATORIA.

MAJOR DOBSON, since the war Senior Medical Officer of the Western Command and proprietor of the Pendyffryn Hall Sanatorium at Penmaenmawr, has been fined (in the person of his wife and one of his patients) for showing a light through open windows, which was visible from the sea. The defence was simply that the institutional rule forbidding closed windows, and the public prohibition of lights, were almost incompatible when a patient was taken ill after dark. The staff, it was pointed out, had great difficulties in attending to patients in the dark, and apparently shaded lights with open windows are virtually not shaded at all. Can anyone suggest a compromise?

### THE DEVON AND CORNWALL HOMŒOPATHIC HOSPITAL.

DETERMINED efforts are to be put forth to relieve the Devon and Cornwall Homœopathic Hospital of the burden of debt left after the extension of the institution years ago. The extension and the furniture cost between £6,000 and £7,000, and in the last eight years over £5,000 has been raised. This year the bank overdraft on the extension account has been reduced from £2,473 to £1,891, but the debt still imposes the necessity of finding over £100 per annum to meet interest and bank charges. A house-to-house canvass of the town for subscriptions is suggested.

### "THE FIFTH" IN FEBRUARY

THE Magazine of the Fifth London (City) General Hospital, known as *The Fifth*, which accomplished its third issue during February, realises, like the most famous of its contemporaries, the value of the small page, of the inset illustration, and the charm produced by similarity of paper throughout. Mr. Charles Dawbarn gives the impressions of a French *poilu* on London: his surprise at any but soldiers being allowed to wear khaki, a permission which would seem "a sacrilege" in France; the gaiety of London and its resources compared with the sadness of Paris. Lady Frances Balfour contributes a poem "found under the pillow of an American soldier who died in hospital during the Civil War," and very good it is. Like most gazettes, it could do with more illustrations, and among its three thousand readers the Editor should not have to wait in vain. We notice in the corresponding issue of the *Southern Cross* a cartoon entitled "Punch-ball," by Pte. James Scott. It is not unworthy of *Punch*, and we hope this draughtsman will meet the encouragement he deserves.

### COPY FROM EVERYWHERE.

THE art of relieving a hospital gazette from the necessarily limited horizon of domestic concerns is best accomplished by cultivating the assistance of ex-patients and former members of the staff. The *Craigleith Hospital Chronicle* accomplishes this better than most of its contemporaries, and we commend to the editors of other gazettes the articles in the March number entitled "A Babe Biography," "Christmas in Salonica," and "Camp Life in Egypt." They instance what can be done in this respect better perhaps than the extraneous contributions of John Buchan and Bertram Smith; on the other hand John Parkinson's article "An Unchartered Company," giving some of his experiences in Southern Nigeria, is welcome for its vivacious reminder of native life on the Kamerun frontier. What wide ground is covered by these articles!

### THIS WEEK'S DRUG MARKET.

THE scarcity of several vegetable drugs is becoming very acute, and although there are few medicinal products of vegetable origin that are actually unobtainable the number of those of which the supply is limited is steadily growing. For instance, there is no grey Jamaica sarsaparilla catalogued for this month's drug sale, while the quantity of gum ammoniacum to be offered is almost negligible. In synthetic drugs there are few changes of importance; acetylsalicylic acid is offered at a further slight reduction; salicylic acid and sodium salicylate are unchanged; phenazone is again slightly dearer; phenacetin is slightly lower in price; it is difficult to find any barbitone except in retail quantities. Cocaine has been offered at something below the highest figure recently quoted, and as supplies are expected to arrive shortly the value will probably recede; nobody should buy more than is necessary for immediate requirements. Acetic acid is again dearer; citric acid and tartaric acid still have an upward price tendency. It is expected that still higher prices will be asked for cod-liver oil. Liquid paraffin has become very scarce again; the stocks of soft paraffin have become greatly reduced and prices have advanced. Caraway oil is very scarce. A little oil of juniper berries is still offered. Glycerophosphates are scarce owing to the difficulty of obtaining raw materials. A further small business has been done in quinine, and the price is well maintained.

